# Retinal Cone Mosaic in *sws1*-Mutant Medaka (*Oryzias latipes*), A Teleost

**DOI:** 10.1167/iovs.63.11.21

**Published:** 2022-10-27

**Authors:** Megumi Matsuo, Makoto Matsuyama, Tomoe Kobayashi, Shinji Kanda, Satoshi Ansai, Taichi Kawakami, Erika Hosokawa, Yutaka Daido, Takehiro G. Kusakabe, Kiyoshi Naruse, Shoji Fukamachi

**Affiliations:** 1 Department of Chemical and Biological Sciences, Japan Women's University, Bunkyo-ku, Tokyo, Japan; 2 Division of Molecular Genetics, Shigei Medical Research Institute, 2117 Yamada, Minami-ku, Okayama, Japan; 3 Laboratory of Physiology, Atmosphere and Ocean Research Institute, The University of Tokyo, Kashiwa, Chiba, Japan; 4 Laboratory of Bioresources/NIBB Center of the Interuniversity Bio-Backup Project, National Institute for Basic Biology, Okazaki, Aichi, Japan; 5 Institute for Integrative Neurobiology and Department of Biology, Graduate School of Natural Science, Konan University, Kobe, Hyogo, Japan

**Keywords:** SWS1 (short wavelength sensitive opsin), LWS (long wavelength sensitive opsin), arrestin, cone photoreceptor, retinal degeneration

## Abstract

**Purpose:**

Ablation of short single cones (SSCs) expressing short-wavelength-sensitive opsin (SWS1) is well analyzed in the field of regenerative retinal cells. In contrast with ablation studies, the phenomena caused by the complete deletion of SWS1 are less well-understood. To assess the effects of SWS1 deficiency on retinal structure, we established and analyzed *sws1*-mutant medaka.

**Methods:**

To visualize SWS1, a monoclonal anti-SWS1 antibody and transgenic reporter fish (*Tg*(*sws1:mem-egfp*)) were generated. We also developed a CRISPR/Cas-driven *sws1*-mutant line. Retinal structure of *sws1* mutant was visualized using anti-SWS1, 1D4, and ZPR1 antibodies and coumarin derivatives and compared with wild type, *Tg*(*sws1:mem-egfp*)*,* and another opsin (*lws*) mutant.

**Results:**

Our rat monoclonal antibody specifically recognized medaka SWS1. S*ws1* mutant retained regularly arranged cone mosaic as *lws* mutant and its SSCs had neither SWS1 nor long wavelength sensitive opsin. Depletion of *sws1* did not affect the expression of long wavelength sensitive opsin, and vice versa. ZPR1 antibody recognized arrestin spread throughout double cones and long single cones in wild-type, transgenic, and *sws1*-mutant lines.

**Conclusions:**

Comparative observation of *sws1*-mutant and wild-type retinas revealed that ZPR1 negativity is not a marker for SSCs with SWS1, but SSCs themselves. Loss of functional *sws1* did not cause retinal degeneration, indicating that *sws1* is not essential for cone mosaic development in medaka. Our two fish lines, one with visualized SWS1 and the other lacking functional SWS1, offer an opportunity to study neural network synapsing with SSCs and to clarify the role of SWS1 in vision.

Animals live in response to their environment. In vertebrates, the eyes play a major role in vision. In the retina of the eye, photoreceptor cells sensitive to various wavelengths convert light stimuli into cellular signals, which are then transmitted to downstream neurons.^1^ Teleost possesses rod cells and four subtypes of cone cells. Typically, rods and cones contain a single visual pigment,^2^^–^^7^ opsin protein bound to a chromophore, which together determines spectral sensitivity.^8^ There are four opsins in cones (short-wavelength-sensitive opsin [SWS]1, SWS2, rhodopsin-like [RH]2, long-wavelength-sensitive opsin [LWS], which are sensitive to UV, blue, green, and red light, respectively) (Fig. 1) and one in rods (RH1). Teleost cone cells form an ordered array with regular spacing, where red and green cones exist as physically fused double cones (DCs).^9^^–^^12^ The blue cone is long with a single outer segment (long single cone [LSC]). The UV cone is short with a single outer segment (short single cone [SSC]). DC, LSC, and SSC formed the crystalline arrays, which are generally categorized into two patterns: row mosaic and square mosaic.^13^^–^^15^ In row mosaics as of zebrafish, double and single cone photoreceptors are arranged in parallel rows.^16^^,^^17^ In other teleost such as goldfish and medaka, retinal cone cells form a lattice arrangement of squares (Fig. 1).^18^^–^^20^ In rainbow trout, these two patterns of cones coexist in the retina.^14^^,^^30^

**Figure 1.**
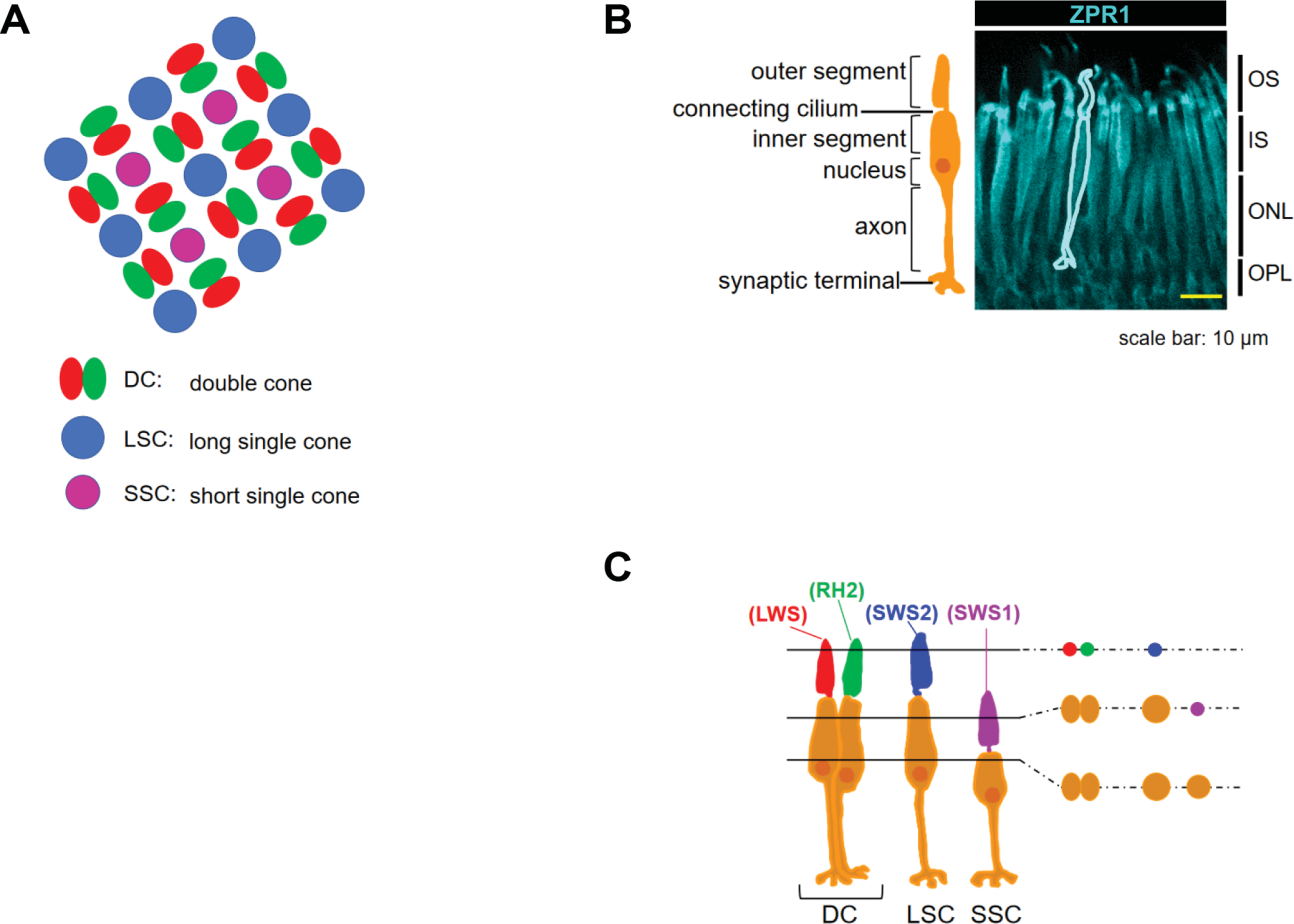
Medaka photoreceptor organization. (**A**) Schematic mosaic arrangement of retinal cone cells of medaka. Medaka has regularly arranged cone mosaic structure in the retina, where DCs (*red* and *green*) and LSC (*blue*) and SSC (*UV*) form a lattice arrangement of squares. SSC is always sandwiched between facing red cones and facing green cones. (**B**) Cone cells visualized with ZPR1 antibody. The fluorescent image is accompanied by a cartoon of a typical cone cell. OS, outer segment of the photoreceptor cells, IS, inner segment of the photoreceptor cells; ONL, outer nuclear layer; OPL, outer plexiform layer. (**C**) Four subtypes of cone cell of medaka. Red and green cone cells (encompassing LWS and RH2 in each outer segment) are paired to form DC. LSC has SWS2 and SSC has SWS1. On the right is a cross-sectional view cut along the line. The outer segments appear as dots and the inner segments as rounds.

In both rod and cone cells, the light activates visual pigments, which subsequently induces a phototransduction cascade.^21^ The termination of light response requires inactivation of light-activated visual pigments by phosphorylation, and visual arrestin completely shuts off the activity of light-activated visual pigments by binding to phosphorylated pigments.^22^^–^^25^ The functional null mutations in *Arrestin* result in a type of congenital stationary night blindness called Oguchi disease.^26^^,^^27^ The visual arrestin is a cytosolic protein with a molecular weight of 40 to 45 kDa, which is encoded by the genes *SAG* (rod arrestin, expressed in rods) and *Arrestin3* (*Arr3*, cone arrestin, expressed in cones).^28^ Fish have two subtypes of *Arr3*, *Arr3a* and *Arr3b*.^29^^–^^32^ Labeling with subtype-specific antibodies (e.g., ZPR1 antibody for Arr3a) reveals subfunctionalized expression of Arr3a in DC and Arr3b in LSC and SSC in zebrafish.^33^^–^^36^

We have used medaka (*Oryzias latipes*), a model animal with a long history of genetic research.^37^^–^^39^ Many established resources, laboratory strains (https://shigen.nig.ac.jp/medaka/top/top.jsp), and techniques used in biological, ecological, behavioral fields are available.^40^^–^^45^ Teleost visual pigments with LWS or SWS1 absorb the longest or shortest parts of wavelength, respectively. Previously, we established *lws*-mutant medaka and showed that decreased red light sensitivity affected behavioral response.^46^^–^^49^ SWS1-expressing SSCs work in prey capture,^50^^,^^51^ but in contrast with *lws*, the effects of the loss of *sws1* have not been well-analyzed, except in mouse and trout. Although a lack of *sws1* in mouse does not cause retinal degeneration,^52^^,^^53^ a recent study of CRISPR/Cas-driven *sws1*-mutant trout reports serious effects on the retina, including retinal degeneration.^54^ Thus, we investigated the effect of *sws1* deficiency on retinal structure and arrestin expression using medaka. To this end, we established two medaka lines, a transgenic *sws1*-reporter and an *sws1*-deficient line, and produced a monoclonal antibody specifically recognizing SWS1.

## Methods

### Medaka Husbandry

We used laboratory-raised, 3- to 12-month-old matured medaka (*O.* *latipes*). Fish were reared under a 14/10-hour light/dark cycle. All the treatments of animals in this research were carried out in accordance with the Japanese Act on Welfare and Management of Animals (Act No. 105 of October 1, 1973; the latest revisions Act No. 51 of June 2, 2017, effective June 1, 2018). All experimental protocols were approved by the Institutional Animal Care and Use Committees of Konan University and by the Animal Experiment Committees of Japan Women's University.

### Establishment of *sws1*-mutant Medaka

**Figure 2.**
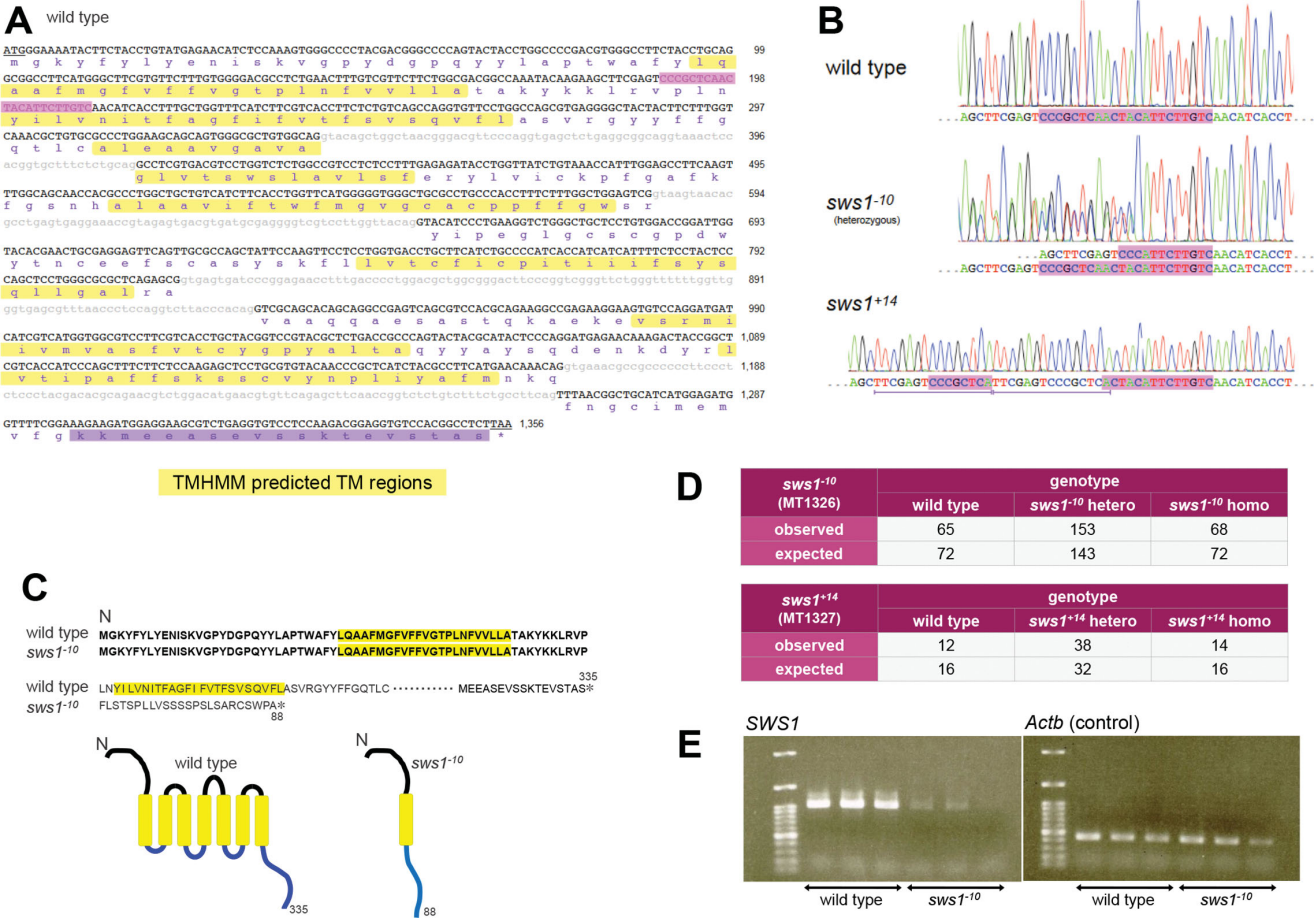
The *sws1*-mutant medaka. (**A**). Genomic and translated peptide sequence of the medaka *SWS1* locus (AB223058). Initiation and termination codons are underlined. Exons and introns are shown as *black* capital and *gray* small letters, respectively. *Magenta* indicates the target sequence for CRISPR/Cas9. The TMHMM predicted transmembrane regions^88^ are highlighted in *yellow*. The peptide sequence used as an antigen for antibody production (see Fig. 3) is highlighted in *purple*. (**B**). Electropherogram of the *sws1* mutations. The target sequence is highlighted in *magenta*. These electropherograms are flipped (i.e., shown as a reverse complement) from the original ones. *Purple* horizontal lines in *sws1^+14^* indicate tandem repeats of 15 nucleotides. (**C**) Deduced SWS1 peptide sequence of *sws1^−^^10^*, aligned with SWS1 of wild type. SWS1 of *sws1^−^^10^* medaka was 88 amino acids long, which has an identical N-terminus to wild-type SWS1 and has the first of seven transmembrane regions of intact SWS1. (**D**) Offspring between the *sws1* heterozygotes. 286 and 64 adults were genotyped for *sws1^−^^10^* and *sws1^+14^*, respectively. Mendelian inheritance should result in a genotype ratio of wild-type:heterozygotes:homozygotes of 1:2:1, which was observed in both mutations (*P* = 0.482 for *sws1^−^^10^* and 0.305 for *sws1^+14^*, χ^2^ test). (**E**) RT-PCR. cDNA synthesized from mRNA in the eye (*n* = 3 each for wild type and *sws1^−^^10^*) was used as a template. Transcript of *SWS1* was decreased in the mutant, whereas that of *actin beta* (*Actb*) was equivalent between the strains.

*Sws1*-mutant medaka was generated with the CRISPR-Cas9 genome editing system (Fig. 2) as described previously.^41^^,^^48^^,^^55^ Primer sets to check the transcripts in the eye were forward: ATGGGAAAATACTTCTACCTGTATGAGAACATC and reverse: TTAAGAGGCCGTGGACACCTCCG for *SWS1* and forward: ATGGATGATGACATTGCCGCACTG and reverse: TTAGAAGCATTTGCGGTGGACGATG for *Actb*. We have established two *sws1*-mutant lines with a 10-bp deletion (*sws1^−^^10^*) and a 14-bp insertion (*sws1^+14^*), both of which caused a frame-shift mutation in exon2 of *SWS1* (Fig. 2). The *sws1^−^^10^* mutant was used in the following analyses.

### Generation of Anti-SWS1 Monoclonal Antibody

**Figure 3.**
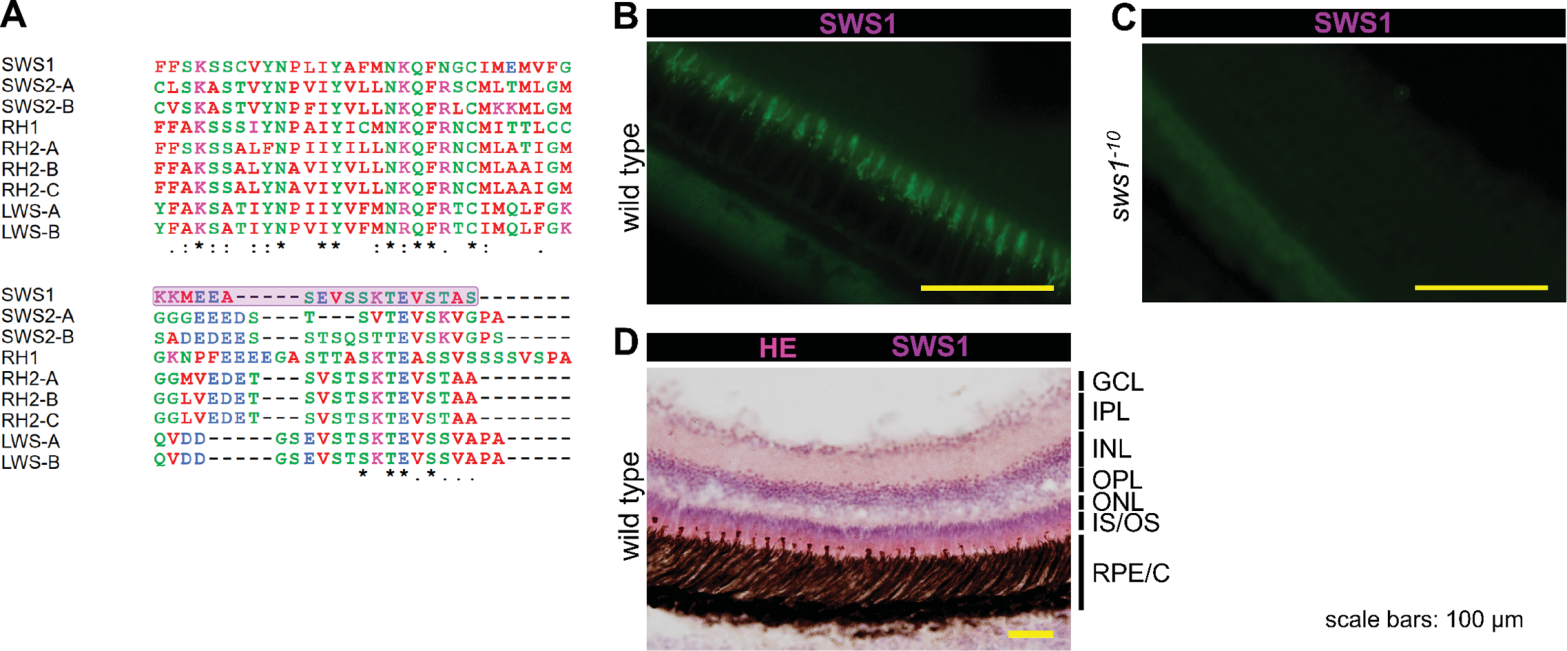
Establishment of an anti-SWS1 monoclonal antibody. (**A**) The peptide sequence of medaka SWS1 used for immunization to rats (*purple*) is aligned with the sequence of other opsins of medaka. The color of amino acid residues means their physicochemical properties. An asterisk (*) indicates a position that has a single, fully conserved residue. A period (.) indicates conservation between groups of weakly similar properties. A colon (:) indicates conservation between groups of strongly similar properties. All the settings are the defaults for Clustal Omega at the European Molecular Biology Laboratory–European Bioinformatics Institute. (**B**, **C**) Scrutiny of medaka SWS1 monoclonal antibody using *sws1*-mutant medaka generated by CRISPR/Cas9 system. (**B**) In wild-type medaka, a characteristic cone-shaped signal was observed in the layer of outer segments of a retina. (**C**) No signal was observed in the retina of *sws1^−^^10^* medaka. (**D**) Immunohistochemical detection of SWS1 in the retina counterstained with hematoxylin and eosin staining (HE). Cone-shaped SWS1 immunoreactive signals were observed in the inner/outer segment (IS/OS) layers. GCL, ganglion cell layer; IPL, inner plexiform layer; INL, inner nuclear layer; RPE/C, retinal pigment epithelium/cells.

Opsin peptide sequences^56^ (for medaka RH1: BAD99136.1) were aligned using CLUSTAL-Omega at the European Molecular Biology Laboratory–European Bioinformatics Institute.^57^ Based on sequence comparison, the C-terminus of SWS1 peptide was chosen as immunogens for generating antibodies (Fig. 3A). We produced rat monoclonal anti-SWS1 antibody, as described previously.^58^^,^^59^ The supernatants of 5 of 39 hybridoma clone cultures were subjected to the immunohistochemical analysis described in this article.

Sexually matured d-rR strain medaka were kept in a dark room for 1 hour to make their melanin granules of pigment epithelium aggregate at the basal region of the cells.^30^ They were then deeply anesthetized with MS-222 (Sigma, St. Louis, MO) and perfused with 4% paraformaldehyde in 0.05 M PBS (pH 7.4) from the conus anteriosus. Retinas were dissected and post-fixed with the same fixative at least 1 hour at 4°C. They were immersed in 30% sucrose in PBS until well-soaked, embedded in 5% agarose (Sigma Type IX) solution containing 20% sucrose, and quickly frozen in cold n-hexane (−60°C). Cross-sections were prepared on slide glasses with a cryostat at 30 µm and dried at room temperature (RT). After penetrating with PBS containing 0.3% Triton X-100, the sections were incubated with one of the supernatants (10% supernatant for fluorescent detection or 2% for 3,3′-diaminobenzidine detection, 5% normal goat serum in PBS) overnight. They were reacted with biotinylated anti-rat IgG (Jackson ImmunoResearch, West Grove, PA) for 1 hour, subsequently with ABC complex (VECTASTAIN ABC kit, vector lab, Burlingame, CA) for 1 hour. Finally, sections were visualized through the fluorescence of Streptavidin, Alexa Fluor 488 (Invitrogen, Waltham, MA) conjugate. In some samples, we performed 3,3′-diaminobenzidine detection followed by counterstaining with hematoxylin and eosin. The supernatant of a hybridoma clone that showed the best signal with low noise was subjected to the specificity check test using wild-type and *sws1^−^^10^* retina with the protocol described elsewhere in this article.

### Establishment of *sws1:mem-egfp* TG Line of Medaka

The 5' flanking sequence of the medaka *SWS1* gene obtained from the Ensembl genome database was used to design gene-specific primers. The *O. latipes* genomic DNA was extracted from one individual of the Hd-rR inbred strain as described previously.^60^ Genomic DNA fragments containing the upstream *cis*-regulatory region of *SWS1* were amplified from the genomic DNA by PCR using a thermostable DNA polymerase (PrimeStar HS DNA polymerase, Takara BIO, Japan) and a pair of gene-specific oligonucleotide primers, 5ʹ-TGACGTCGACTCTGGTTCTGGTCCTG-3ʹ and 5ʹ-ACGGATCCGTGAAGCTGAGCTCTG-3ʹ.^61^ The pBluescript-mem-EGFP vector was made by inserting the oligonucleotides corresponding with the membrane-anchoring signal of neuromodulin (5ʹ-atgctgtgctgtatgagaagaaccaaacaggttgaaaagaatgatgaggaccaaaagatc-3ʹ)^62^ into the 5ʹ end of the EGFP-coding region of pBluescript-EGFP,^63^ generating an open reading frame encoding a fusion protein of the N-terminal 20 amino acids of neuromodulin and EGFP that can label plasma and intracellular membranes with fluorescence.^64^ The amplified 1.2-kb upstream region of *SWS1* was inserted into the *Sal*I/*Bam*HI sites of pBluescript-mem-EGFP. The resultant plasmid DNA was introduced into medaka embryos by microinjection as described previously.^65^ The injected embryos were screened for fluorescence in retinal photoreceptor cells under a fluorescent dissection microscope (M165 FC; Leica Microsystems, Wetzlar, Germany) and then reared to adult fish. The transgenic strain *Tg*(*sws1:mem-egfp*) was established by successive crosses and selection of the fish with green fluorescent protein (GFP) fluorescence in retinal photoreceptor cells. In immunohistological analyses, for double labeling of the *Tg*(*sws1: mem-egfp*) retina with GFP and anti-SWS1 or ZPR1 (Abcam, Cambridge, UK), GFP was visualized by immunostaining with rabbit anti-GFP polyclonal antibody (Invitrogen) and Alexa Fluor 488-conjugated anti-rabbit IgG secondary antibody, diluted 1000-fold. Anti-SWS1 and ZPR1 signals were detected with Alexa Fluor 594-conjugated secondary antibodies (Invitrogen, for details of immunohistological procedures, see the Histology of Whole-mount Retina section).

### Histology of Whole-mount Retina

The whole retina was isolated from the eyecup of dark-adapted medaka according to the method of Salbreux et al.,^66^ with some modification. The retina was flushed out from the enucleated eye and rinsed in ice-chilled PBS. After the isolation and fixation in 4% paraformaldehyde in 0.1 M phosphate buffer (pH 7.4) with 5% sucrose for 15 minutes twice and 30 minutes once, the retina was transferred onto nonfluorescent adhesive glass slides (MAS-coated glass slides, Matsunami Glass Ind., Osaka, Japan). The fixed retina was rinsed three times with 0.1M phosphate buffer containing 5% sucrose every 20 minutes and then once with PBST (PBS with 1% Tween, 1% Triton X-100, and 1% DMSO) for 15 minutes. After rinsing, the retina was blocked in 10% normal rabbit serum at RT for 1 hour, treated with anti-SWS1 antibody diluted 10 times by PBST, covered with dice-size-cut parafilm in a moist chamber, and incubated at RT for more than 14 hours. The specimen was washed twice with PBST for 10 minutes, then once with PBS for 5 minutes. These washing steps were conducted routinely between each step. After 2 hours of incubation with biotinylated anti-rat IgG, followed by ABC reagents for 30 minutes, Alexa Fluor 555-conjugated streptavidin (1/1000) was reacted for 30 minutes at RT. Finally, the retina was coverslipped with ProLong Gold (Invitrogen).

For immunocytochemistry with ZPR1 or 1D4 (ab5417, Abcam, UK), each antibody in PBS (1/1000) with 5% normal rabbit serum was reacted at RT for more than 14 hours. The retina was incubated in PBS with rabbit anti-mouse IgG conjugated with Alexa Fluor 633 (1/500, Invitrogen) for 1 hour at RT. We injected 3-(2-Benzothiazolyl)-7-(diethylamino)-coumarin (BTDEC, also known as coumarin 6; Tokyo Chemical Ind., Tokyo, Japan) into a secondary antibody solution (1 µg/mL) to label the retinal cell. Positively stained structures were pseudocolored in orange. For triple labeling using anti-SWS1, ZPR1 or 1D4, and BTDEC, a binding step of anti-SWS1 followed the incubation with ZPR1 or 1D4. Positive signals of anti-SWS1, 1D4, and ZPR1 were pseudocolored in violet, white, and cyan, respectively.

### Imaging of Whole-mount Retina

The retinal images were recorded sequentially using Olympus FV1200 Laser Scanning Confocal Microscope (Olympus, Tokyo, Japan). Image stacks with 0.1 to 2.0 µm step depths were processed with FIJI software.^67^^,^^68^ Starting from the outer plexiform layer, z-depth increased toward the RPE.

## Results

### Establishment of *sws1*-mutant Line and Anti-SWS1 Monoclonal Antibody

We previously established colorblind medaka by knocking out the *cone-opsin* genes using CRISPR/Cas9 technology.^48^^,^^55^ Using the same protocol but different guide RNA, we introduced frameshift mutations in the *SWS1* gene. The target sequence existed in the first exon coding the first transmembrane domain, and a codon for the second methionine was found in the second exon coding the fourth transmembrane domain (Fig. 2A). The frameshift mutations were a ten-base deletion (*sws1^−^^10^*) or a 14-base insertion (*sws1^+14^*; Fig. 2B). The *sws1^+14^* mutation had 15-base tandem repeats, which might reflect an error in the microhomology-mediated end joining. Opsins fold into a seven-transmembrane structure, typical of G protein-coupled receptors,^56^^,^^69^ but SWS1 of *sws1^−^^10^* had only the first transmembrane region and was no longer a G protein-coupled receptor. Therefore, no functional protein could be translated from the mutated allele (Fig. 2C).

Heterozygotes or homozygotes of the *sws1* mutant were indistinguishable from wild type by appearance and seemed to be fully viable under our breeding condition (Fig. 2C) (*P* > 0.05, χ^2^ test). However, fertilized eggs could hardly be obtained from the *sws1^+14^* homozygotes for an unknown reason; inbreeding depression might occur because of the limited number of offspring, and we gave up maintaining *sws1^+14^* fish. Frozen sperms of the *sws1^−^^10^* and *sws1^+14^* mutants are available at NBRP medaka as MT1326 and MT1327, respectively. Transcripts of *sws1* were greatly reduced in the eyes of *sws1^−^^10^* fish, likely reflecting nonsense-mediated mRNA decay (Fig. 2D). Our rat anti-SWS1 monoclonal antibody showed characteristic cone-shaped outer segments of cone cells in wild-type retina, but not in *sws1^−^^10^* (Fig. 3).

### Retinal Cone Mosaic of *Tg*(*sws1:mem-egfp*) Medaka

**Figure 4.**
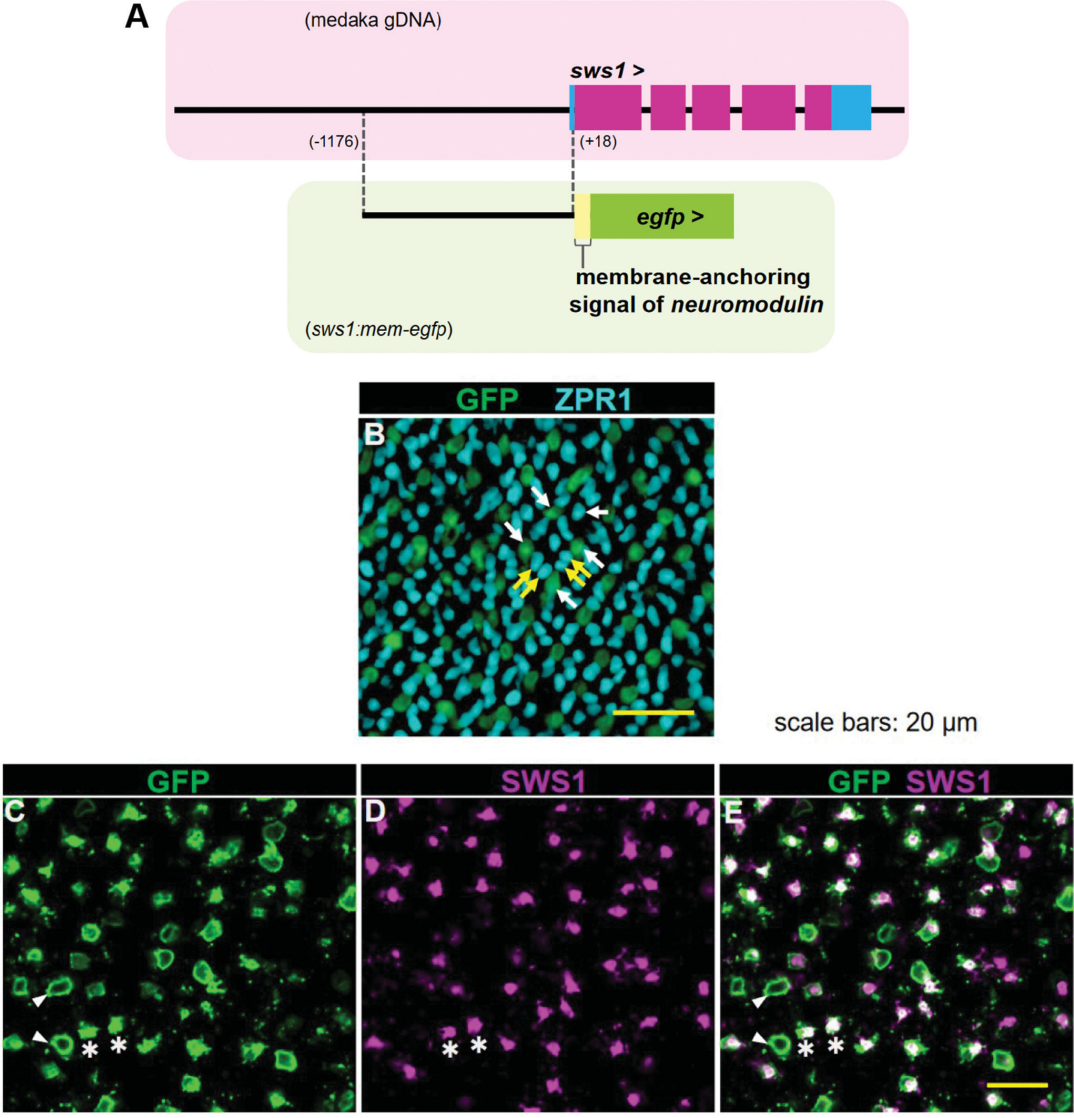
Retinal cones of *Tg*(*sws1:mem-egfp*) medaka. (**A**) Schematic diagram showing the reporter construct of *sws1:mem-egfp*. Numbers in parentheses indicate the nucleotide positions relative to the transcription start site of *sws1*. And position (+19) is the translation start position of *sws1*. The upstream region of *sws1* was fused with the membrane-anchoring signal of neuromodulin and EGFP. (**B**–**D**) Cone mosaic arrangement in *Tg*(*sws1:mem-egfp*) medaka's retina. (**B**) Cells that expressed *sws1* were visualized through the anti-GFP antibody. ZPR1 detected cells without anti-GFP labeling, LSCs and both members of DCs. An anti–GFP-positive cell was surrounded by ZPR1-positive cone cells, and both together made up a square mosaic. *White arrows* point to single cones, and *yellow arrows* point to DCs. (**C**) Anti-GFP signals were observed as *green rounds* and *dots*, that is, the inner segments and outer segments, respectively. (**D**) Anti-SWS1 signals were observed as *violet dots*, that is, outer segments. (**D**) Merged image of (**B**) and (**C**). *Green dots* of anti-GFP and *violet dots* of anti-SWS1 colocalized, showing that the outer segment with SWS1 was detected by a monoclonal antibody against SWS1. (**B**–**D**) *White arrowheads* point to anti–GFP-labeled *green rounds*. *Asterisks* point to cells in which both anti-GFP and anti-SWS1 signals overlapped.

We visualized cone cells expressing *sws1* by generating a transgenic line, *Tg*(*sws1:mem-egfp*). The 1.2-kb upstream region of medaka *sws1* containing *cis*-regulatory sequences^61^ was sufficient to drive reporter expression in cone cells (Fig. 4B, C). When a cone expressing *sws1* and having one protrusion was visualized with an anti-GFP antibody, its inner segment appeared as a green round and its outer segment as a green dot (Fig. 4C). Double immunolabeling of flat-mounted *Tg*(*sws1:mem-egfp*) retina with anti-GFP and ZPR1 antibodies revealed cone mosaics where a single cone subtype had GFP, and the other cone subtypes were labeled by ZPR1 (Fig. 4B). GFP-positive and ZPR1-positive cones together constituted square mosaics. When *Tg*(*sws1:mem-egfp*) retina was reacted with anti-SWS1 and anti-GFP antibodies, two signals colocalized in the outer segment of single cones but did not overlap in the inner parts of cells (Fig. 4C–E, Supplementary Video S1), suggesting that anti-SWS1 antibody specifically recognized the outer segments of single cones expressing *sws1*.

### Square Cone Mosaic Visualized Using Anti-SWS1, ZPR1 Antibodies, and Coumarin in Wild-type Medaka

**Figure 5.**
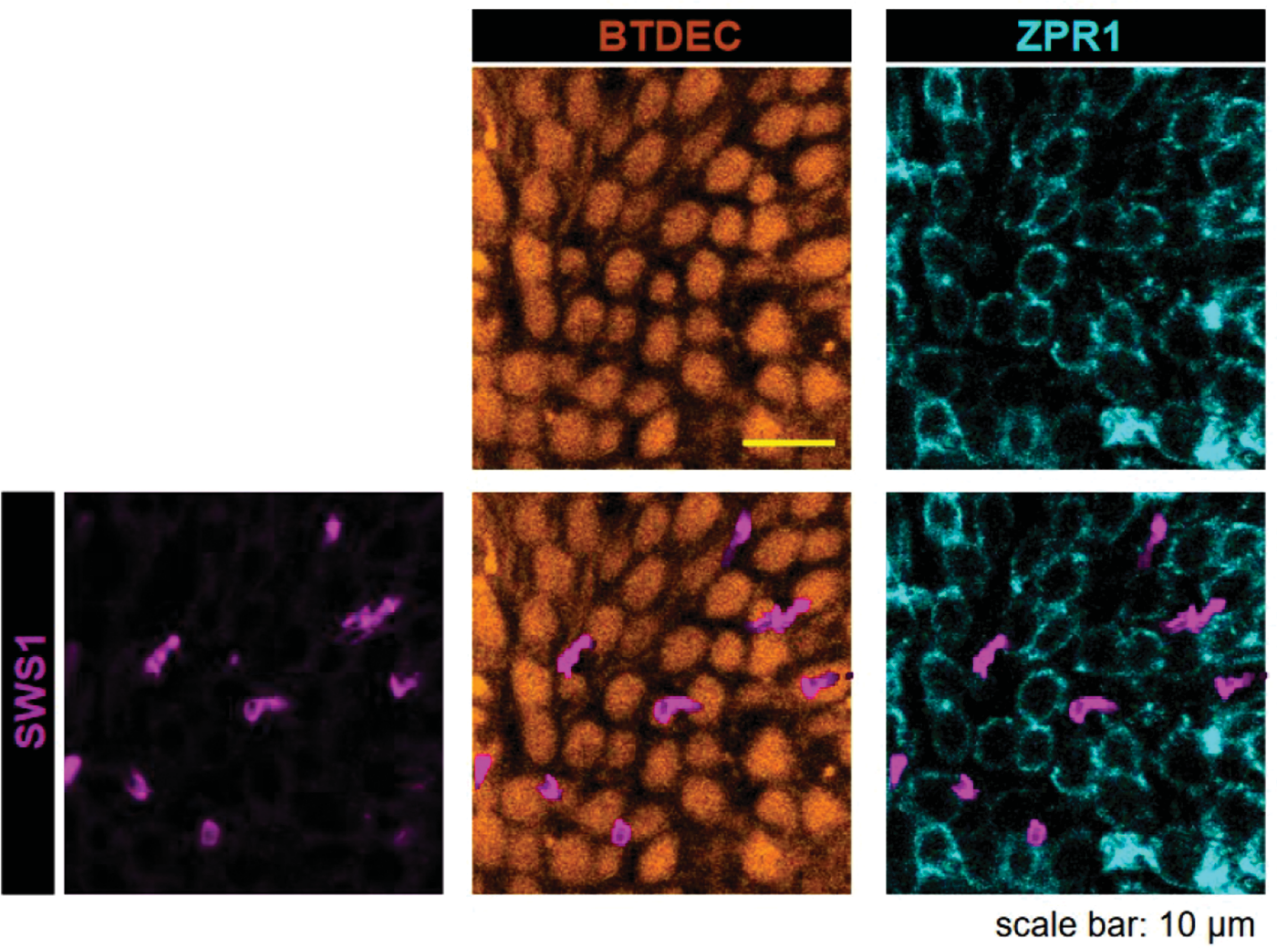
Retinal cones of wild-type medaka. Photoreceptor cells in medaka's retina were visualized with ZPR1 (*cyan*) and BTDEC (*orange*) in the *upper panel*, and with anti-SWS1 (*violet*), ZPR1 antibodies, and BTDEC in the *lower panel*. BTDEC showed the regular arrangement of cone cells where the ZPR1 signal was absent in one cone subtype. *Violet* anti-SWS1 signal existed in the ZPR1-vacant space, showing that the cone cell expressing SWS1 was ZPR1-negative.

**Figure 6.**
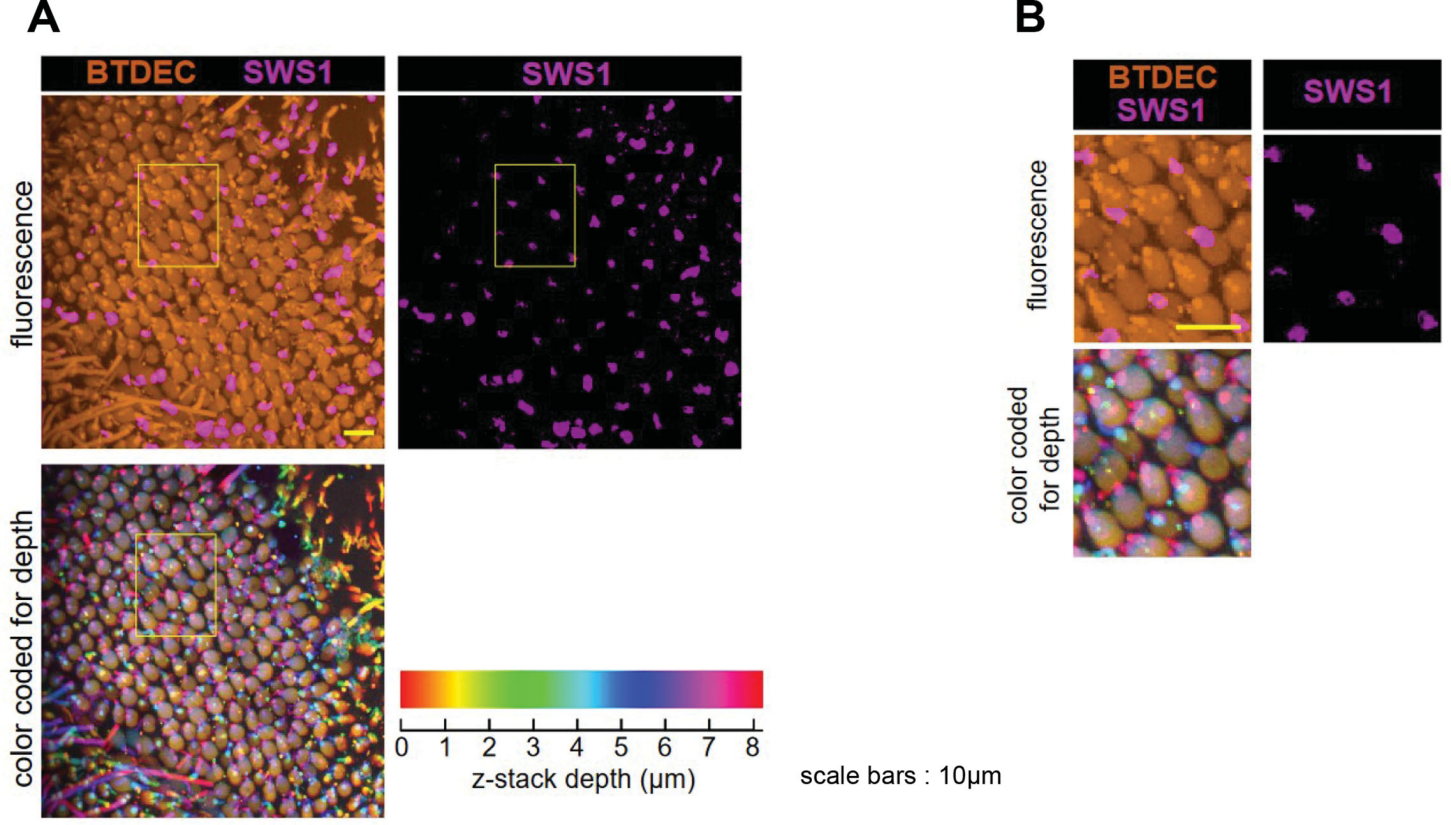
Volume-rendered z-stacks of the retinal photoreceptor cells of wild-type medaka. (**A**) Photoreceptor cells visualized by BTDEC and anti-SWS1 antibody. Shown are fluorescent images (*upper panel*) and z-depth stacks color-coded for depth (*lower panel*). BTDEC visualized rod photoreceptor cells and regularly arranged cone cells. The Inner segments of cones were observed at deeper positions than the outer segments. (**B**) A *yellow boxed* retinal area in (**A**) shown at higher magnification. A single protruding outer segment with SWS1 was *blue* in z-stacks. The cone with SWS1 was surrounded by other cones whose outer segments were *magenta* to *red*. Thus, cone cells expressing SWS1 were shorter than other cone subtypes.

Next, we validated an arrangement of retinal cones in wild-type medaka, using coumarin derivatives and antibodies. Because coumarin stains retinal cells in zebrafish,^70^ we used BTDEC for immunohistochemistry of the whole mounted retina of medaka. BTDEC visualized cone cells spread over the retina (Fig. 5), where fluorescence was strong in the outer segment and somewhat weak in the inner segment of cones (Fig. 6). ZPR1 visualized cone photoreceptors, from the tip of the outer segment to the inner segment, axon, and synaptic terminal (Fig. 1). The retina with ZPR1 and anti-SWS1 antibodies showed that ZPR1-negative cones had SWS1. ZPR1-positive cones and anti–SWS1-positive cones together made up square mosaics (Fig. 5).

To confirm the retinal cell arrangement in detail, such as photoreceptor subtypes, we built a 3D model of acquired sequential images. As seen in the z-stacks in Figure 6, BTDEC visualized regularly arranged cones. The SWS1-positive protrusions were blue in color-coded stacks for depth, whereas those of surrounding cones were magenta to red (Fig. 6). Hence, SWS1-positive cones were shorter than other cone subtypes. In summary, anti-SWS1 monoclonal antibody detected the outer segment of SSC in wild-type medaka.

### Retained Regularity of Cone Arrangement in *sws1^−^^10^* Medaka's Retina

**Figure 7.**
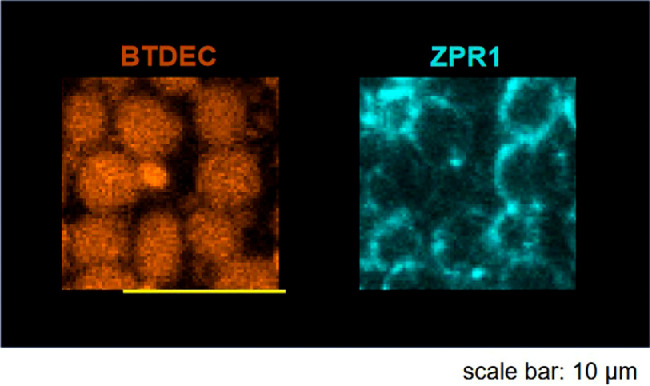
Retinal cones of *sws1^−^^10^* medaka. A single dot-shaped outer segment of a ZPR1-negative cell was surrounded by round-shaped inner segments of other cones.

To assess the effects of SWS1-depletion on retinal structure, we analyzed *sws1^−^^10^* medaka using the same strategies as transgenic and wild-type medaka. The *sws^−^^10^* medaka lost SWS1-signal in the retina, where BTDEC-stained cones formed regular arrangement (Fig. 7) just as seen in wild-type medaka (Fig. 5). At depths where a single outer segment of a ZPR1-negative cell appeared, inner segments of other cone subtypes were observed as orange rounds (Fig. 7).

**Figure 8.**
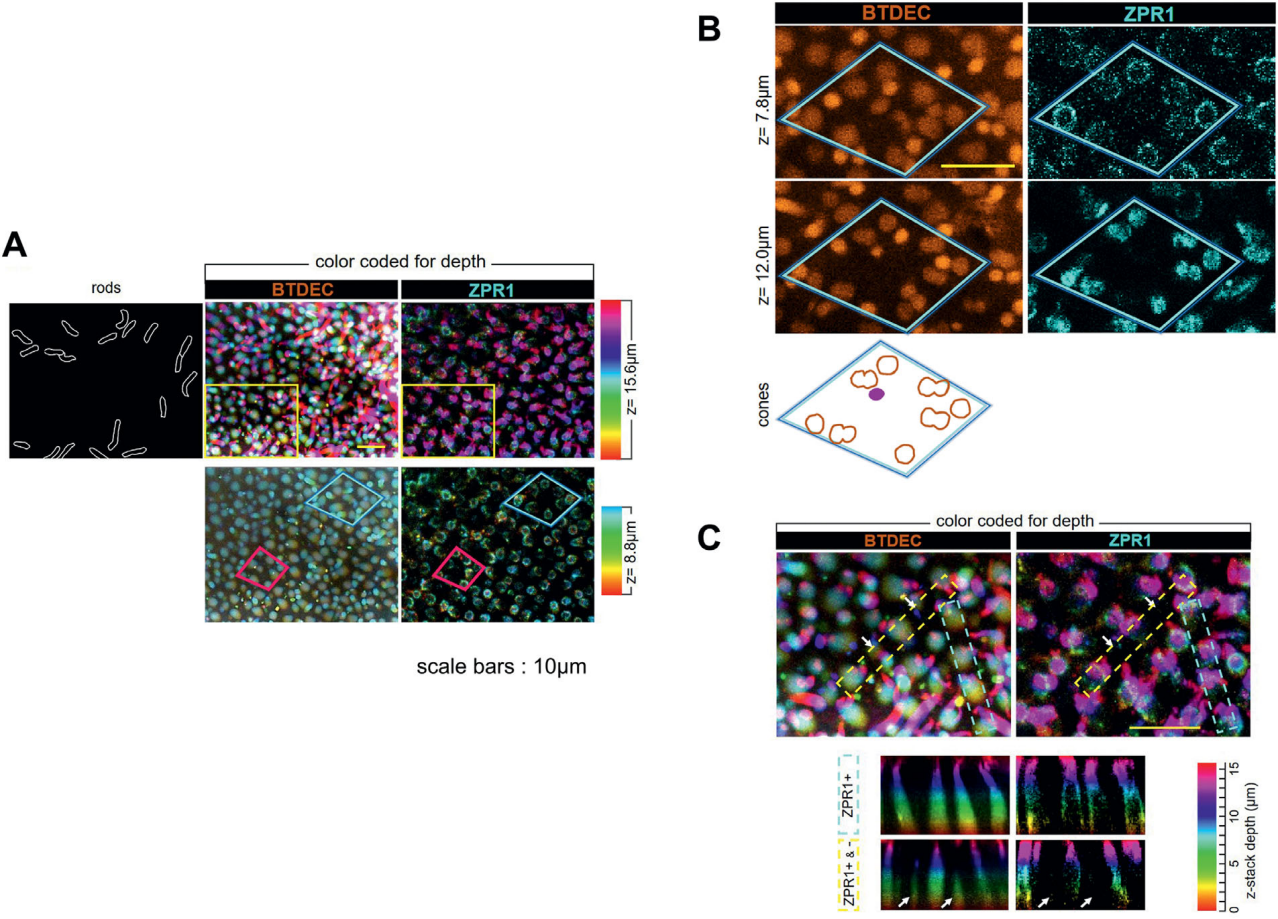
Volume-rendered z-stacks of the retinal photoreceptor cells of *sws1^−^^10^* medaka. Photoreceptor cells were visualized with BTDEC and ZPR1. Stacks were color coded for depth. (**A**) The z-stacks of z = 0 to 15.6 µm in the *upper panel*, and the z-stacks of z = 0 to 8.8 µm in the *lower panel*. BTDEC stained rod cells as well as cones. In the *upper panel*, a schematic of rods is displayed on the left side of the BTDEC image. ZPR1 did not label rod cells. In the *lower panel*, cones formed regular arrangements (e.g., see areas boxed by *blue* and *red quadrangles*). In the space where there was no ZPR1 signal, cells did not disappear but existed. (**B**) A *blue boxed* retinal area in (**A**) at higher magnification. Shown are fluorescent images at depths of 7.8 µm (*top panel*) and 12.0 µm (*middle panel*) at the identical *x*–*y* coordinates. In the *top panel*, a square mosaic appeared, and in the *middle panel*, DCs and single cones were obvious. At the bottom of the figure, a schematic of cone mosaics is depicted. A ZPR1-negative cell (*violet*) with one protrusion was surrounded by other cone subtypes. (**C**) A *yellow boxed* retinal area in (**A**) shown at higher magnification. In the *upper panel*, BTDEC visualized regularly arranged cones with or without ZPR1 signal. Orthogonal views of the retinal area surrounded by *yellow dotted* and *light blue dotted rectangles* in the *upper panel* were displayed in the *lower panel*. The outer segments of all ZPR1-positive cells were *red*. Those of ZPR1-negative cells were *green* to *blue*, indicating shorter than ZPR1-positive cells. *White arrows* point ZPR1-negative cells.

A z-depth stack model visualized retinal cells of *sws1^−^^10^* medaka; BTDEC stained both rods and cones, whereas ZPR1 stained cones (Fig. 8A). The stacks of z = 0 to 15.6 µm with BTDEC and ZPR1 showed the outer and inner segments of cones. The stacks of z = 0 to 8.8 µm exhibited the inner segments, forming square mosaics (Fig. 8A). Higher magnified images represented that the outer segment of a ZPR1-negative cell was surrounded by the inner segments of four DC cells and four single cone cells (Fig. 8B). An orthogonal view of the z-stacks of BTDEC and ZPR1 showed the entire cone (Fig. 8C). All cones were visualized with BTDEC, regardless of ZPR1 signal. ZPR1-negative cones were shorter than the surrounding cones (Fig. 8C). Comprehensively considering Figures 7 and 8, in *sws1^−^^10^*, ZPR1-negative cells were SSCs. Overall, *sws1^−^^10^* medaka retained regular cone mosaic arrangements just as wild type. Even though *sws1^−^^10^* did not express SWS1, SSC was not lost. Furthermore, in *sws1^−^^10^* medaka's retina, ZPR1 labeled both DC and LSC, but not SSC.

**Figure 9.**
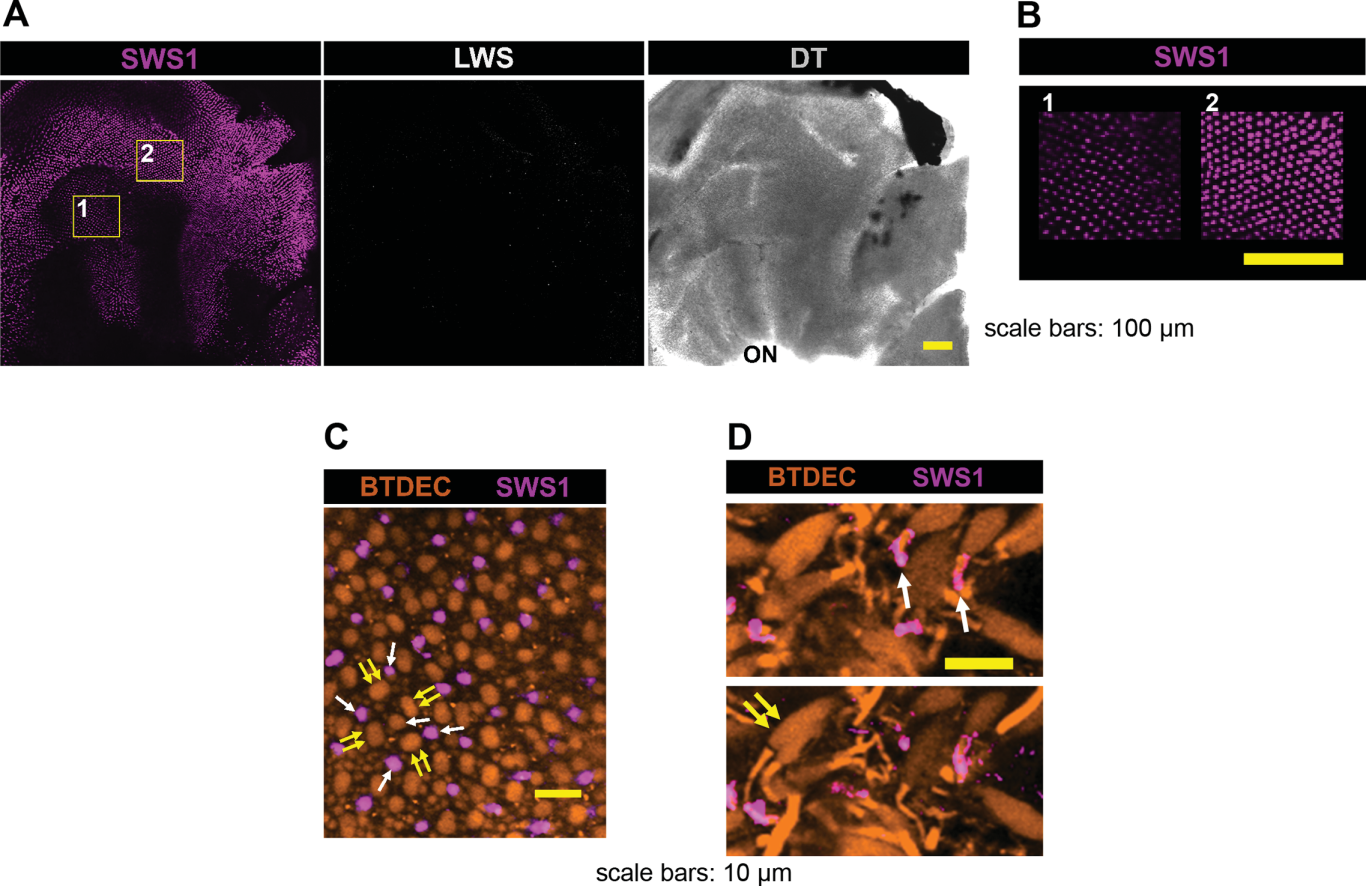
Retinal cones of *lws^+2a+5b^* medaka. (**A**) An isolated retina observed under low magnification. Positively labeled cells with anti-SWS1 antibody spread over the retina, but no signal was obtained with 1D4 antibody. DT, light field image by differential interference observation; ON, optic nerve. (**B**) Two boxed areas in (**A**) observed under high magnification. *Violet* cells with anti-SWS1 antibody spread with regular spacing. (**C**) A retinal mosaic arrangement of cones. Only single cones were positively labeled with anti-SWS1 antibody. (**D**) Two images at identical *x*–*y* coordinates, but different depths. The outer segments of single cone cells had anti-SWS1 signal (*upper panel*), but DCs were not labeled by anti-SWS1 antibody (*lower panel*; 1.4 µm closer to the RPE than the *upper panel*). The *yellow arrows* indicate DCs, and *white arrows* indicate single cone cells.

**Figure 10.**
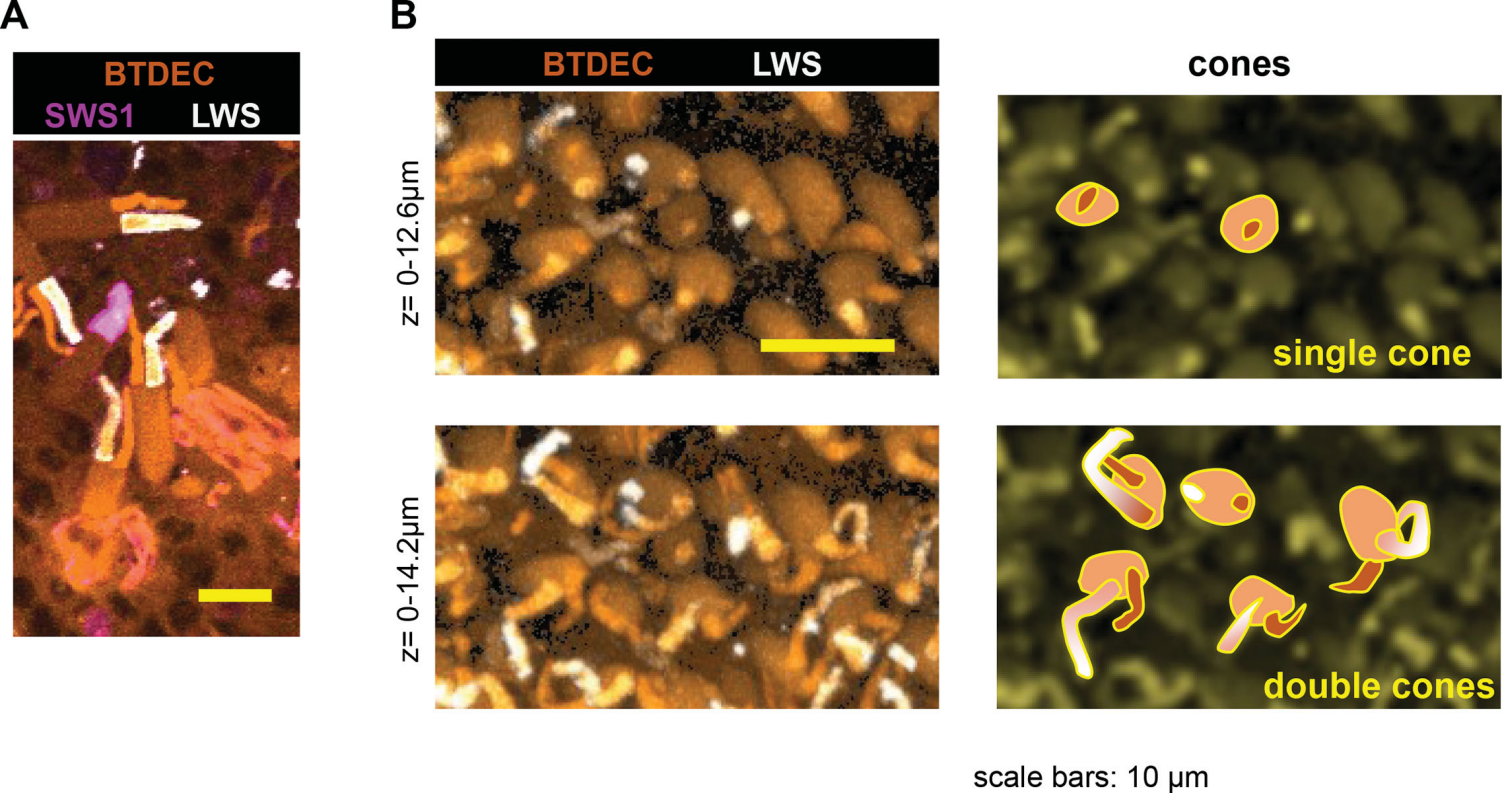
Retinal cones of wild-type and *sws1^−^^10^* medaka. (**A**) Photoreceptor cells visualized by anti-SWS1, 1D4 antibodies and BTDEC in wild-type medaka. When a retina was rinsed well with PBS, few cones remained on the retina and each cone cell was easily identified. Anti-SWS1 antibody bound to a single cone cell, and 1D4 antibody did to one of DCs. (**B**) Z-stack images of retinal cones of *sws1^−^^10^* medaka. Shown are the z-stacks of z = 0 to 12.6 µm in the *upper panel*, and the z-stacks of z = 0 to 14.2 µm in the *lower panel*, accompanied by cartoons of cones on the *right side*. One member of DCs was labeled with 1D4 antibody, and the single cones were not.

Next, we analyzed previously established another *opsin-gene* mutant, *lws^+2a+5b^* line,^48^ to compare the effect of opsin depletion on retinal development. Anti-SWS1 antibody visualized cells with regular spacing, and BTDEC merged mosaic arrangement of cones in *lws^+2a+5b^* retina (Fig. 9). The 1D4 antibody binds to bovine rhodopsin,^71^ but in zebrafish, it labels LWS-expressing cells,^34^ and is used as a red cone marker.^72^ In wild-type medaka, 1D4 labeled the outer segment of one member of DC (Fig. 10A). The 1D4 signals disappeared in *lws^+2a+5b^* medaka (Fig. 9), revealing that it bound to LWS in DCs of medaka. Anti-SWS1 antibody labeled single cone of *lws^+2a+5b^* but not DCs (Fig. 9), and 1D4 conversely labeled one of the DCs in *sws1^−^^10^* but not SSC (Fig. 10B). *Sws1* depletion had a negligible effect on *lws* expression and vice versa. In summary, neither retinal cone mosaic nor ZPR1- or 1D4-positive cells changed in *sws1^−^^10^*.

## Discussion

ZPR1 antibody binds to zebrafish Arr3a in DCs,^33^^–^^36^ which is orthologous to medaka *Arr3a* in DC and LSC.^31^ Given that DCs and LSCs were ZPR1 positive in this study, ZPR1 detected Arr3a in medaka. SSCs did not possess Arr3a, whether they expressed SWS1 or not. Thus, Arr3a negativity is a marker for SSCs, not for cones expressing SWS1. In zebrafish retina, the expression of arrestin is partitioned among cone subtypes; DCs express Arr3a, whereas LSCs and SSCs express Arr3b.^29^ Contrasting zebrafish LSC, which possess Arr3b, medaka LSC had Arr3a. Whereas zebrafish and medaka have regularly arranged cones, LSCs of these two teleost species differ in the position of their mosaics, which may be related to the fact that the LSCs of these two species express different arrestin subtypes.

Mutations in blue, green, and red opsin and rhodopsin genes cause eye disorders and affect visual ability in humans.^73^^–^^75^ So far, animal models have been analyzed, indicating that the effects of mutations in opsin genes are not canonical and are diverse.^76^ For instance, in mice with a targeted disruption of rhodopsin, not only rods but also cones degenerate, and the outer segments of cones almost completely disappear.^77^ In zebrafish, meanwhile, mutations in rhodopsin gene cause rod degeneration, but cones are unaffected.^78^ In mice having mutations in M-opsin, M-opsin–dominant cones remain viable for at least 15 months, albeit with shortened or no outer segments, whereas S-opsin–dominant cones are normal.^79^^,^^80^ Given this finding, it would be interesting to know what opsins are expressed in the SSC of *sws1^−^^10^* and DC of *lws^+2a+5b^* medaka, both of which had neither SWS1 nor LWS. Besides histological observation, we assessed the behavioral phenotypes of *sws1*-mutant (SF, in preparation). Previously, we showed that *lws*- or *sws2*-mutant had reduced body color preferences.^49^ According to optomotor response, *lws*-mutant decreased red-light sensitivity^46^^,^^48^; however, blue light sensitivity did not change in *sws2*-mutant.^55^ We conducted mate choice and optomotor response assay of *sws1*-mutant, but their body color preference and UV sensitivity were equivalent to wild type. Furthermore, *sws1*/*sws2*–double-mutant had the same UV sensitivity as wild type. Considering these behavioral results, a new assay needs to be developed to assess the potential defect caused by the loss of *SWS1* in medaka.

As mentioned elsewhere in this article, zebrafish is another well-studied fish model, but an *sws1-*mutant line has not been reported. Instead, ablation studies are available. Ablating zebrafish SSCs (expressing SWS1) stimulates a regenerative response.^81^^–^^83^ Zebrafish H3 horizontal cells exclusively connect to SSCs. When SSCs were ablated, H3 horizontal cells prioritize wiring with SSCs. However, when regeneration of SSCs is delayed or absent, H3 cells increase connections with LSC and DCs.^82^^,^^84^ It is unclear whether the morphology of SSCs or SWS1 of SSCs is important for horizontal cells to prefer SSCs. Elucidating the neural network postsynaptic to SSC of the *sws1^−^^10^* retina will give us a clue to this question and suggest a role of SSCs without SWS1 in vision. So far, *sws1*-deficient animal models have been established in mouse and trout. The lack of *sws1* does not cause retinal degeneration in mouse,^52^^,^^53^ but causes serious retinal developmental defects in rainbow trout.^54^ *Sws1* deficiency did not lead to retinal degeneration in medaka. The reason why the loss of *sws1* did not induce serious effects as trout is unclear, but the sequence of opsin genesis may explain it. Lateral induction mechanisms have been anticipated as causal in creating fish cone mosaics in cyprinid fishes (goldfish and zebrafish),^16^^,^^17^^,^^85^ where the order of cone opsin appearance is LWS, followed by RH2, SWS1, and SWS2.^5^^,^^7^^,^^8^^,^^86^ In contrast, in situ hybridization results indicate that in salmonids, SWS1 appears first, followed by LWS, RH2, and SWS2.^87^ As per the lateral induction mechanism, defects of cone subtype induced early in retinal development would have a significant impact on the developing retina, whereas defects of cone subtype induced later would have little effect. Because SWS1 is the first opsin induced in trout, this mechanism sounds acceptable. If so, in medaka, SWS1 must be induced later in the developing retina, because SWS1 depletion did not affect retinal cone mosaics. Another interpretation of this study is that SWS1 is only a marker for wild-type SSCs in medaka. Because *lws^+2a+5b^* also retained its mosaic structure, opsins may not have a significant effect on retinal development in medaka. However, to determine whether these two hypotheses are plausible, further experiments are needed, such as elucidating the order of opsin genesis in medaka and analyzing the retinal structure of other opsin gene mutants.

## Conclusions

Overall, we provided a monoclonal antibody specifically binding to medaka SWS1, *sws1*-reporter TG (*Tg*(*sws1:mem-egfp*)) line, and *sws1*-mutant line of medaka. Arr3a negativity was not a marker of cones with SWS1, but SSCs. Depletion of *sws1* or *lws* did not cause retinal degeneration and did not affect each other's expression. Our two fish models visualizing *sws1* or lacking functional *sws1* offer an opportunity to analyze the role of SSC in vision and neural networks postsynaptic to SSC.

## Supplementary Material

Supplement 1

## Supplementary Material

**Supplementary Video**. The whole mounted retina of *Tg*(*sws1:mem-egfp*) medaka labeled with anti-GFP (*green*) and anti-SWS1 (pseudocolored *violet*) antibodies. The synaptic terminal of a cone cell appeared first, followed by the axon, the inner segment, and finally, the outer segment.
